# Evaluation of protein C and S levels in patients with COVID-19 infection and their relation to disease severity

**DOI:** 10.1186/s43162-023-00195-3

**Published:** 2023-02-20

**Authors:** Ahmed Elshafie, Enas Foda, Mahmoud M. G. Yousef, Kareem A. Abd El-Naby

**Affiliations:** 1 Internal Medicine Department, Hepatology and Gastroenterology Unit, Faculty of Medicine, Ain Shams University, Cairo, Egypt; 2 Internal Medicine Department, Armed Forces College of Medicine, Cairo, Egypt

**Keywords:** COVID-19, Protein C, Protein S, Thromboembolism

## Abstract

**Background:**

The COVID-19 pandemic has been associated with millions of deaths around the world. One of the important causes of death associated with COVID-19 was pulmonary thromboembolism. The risk for venous thromboembolism was markedly increased in patients with COVID-19 especially those admitted to the intensive care unit. The aims of our study were to measure the protein C and S levels in COVID-19-infected patients in comparison with the normal population and to assess the correlation of protein C and S levels in the plasma to the severity of infection.

**Methods:**

This was a case–control study measuring the protein C and S levels in patients infected with COVID-19 at the time of diagnosis compared to the normal population. The study included one hundred participants, sixty of them are patients with COVID-19, and forty of them are normal healthy adults. The patient group was subclassified into three subgroups according to disease severity: mild, moderate, and severe COVID-19 infections.

**Results:**

The activity of protein C in the patient group serum was significantly lower than that in the control group serum (79.35 ± 26.017 vs 97.43 ± 15.007; *p* < *0.001*). Protein S is also significantly decreased in patients’ serum when compared to the control group (70.233 ± 22.476 vs 91 ± 14.498; *p* < *0.001*). There was a statistically significant decrease in the levels of protein C and S associated with the increase in disease severity (*p* < 0.05). However, protein S showed no statistically significant difference between the moderate and severe disease subgroups.

**Conclusion:**

The study concluded that the levels of protein C and S activities were both decreased in patients with COVID-19 when compared to the healthy population. It also concluded that the decrease in their levels is statistically significant in relation to the disease severity.

## Background

COVID-19 has caused a global outbreak starting in December 2019 in China [1]. Since then, it caused a global pandemic with confirmed infection in more than 600 million patients worldwide and over 6.5 million deaths [2]. In Egypt, above 500,000 were infected with COVID-19 and nearly 24,000 deaths [2].

The COVID-19 infection may be asymptomatic. Fever, fatigue, and cough are typical symptoms in mild COVID-19 cases. The clinical condition may progress to a severe form of interstitial pneumonia which may be deteriorated to respiratory failure, ARDS, and death [3].

COVID-19 infection is associated with a state of hypercoagulability which is called COVID-19-associated coagulopathy (CAC) [4]. The risk for venous thromboembolism (VTE) was markedly increased, especially in patients admitted to the intensive care unit (ICU), with early case series reporting a prevalence of 25 to 43 percent in ICU patients [5]. Pulmonary thromboembolism is one of the most important causes of death in patients with COVID-19 after respiratory failure and bacterial pneumonia [6].

However, the underlying mechanisms leading to CAC are unclear. This state is associated with acute inflammatory changes and laboratory findings that are distinct from acute disseminated intravascular coagulation (DIC) [7]. Evidence supports that the CAC mechanism may be due to the complex interactions between the innate immune response, the coagulation cascade, fibrinolytic pathways, and the vascular endothelium, resulting in a procoagulant condition [8].

Proteins C and S play an important role as anticoagulants by inactivation of coagulation factors Va and VIIIa [9]. Decreased levels of these proteins are associated with an increased risk of venous thromboembolism [10]. Thus, this study aimed to assess serum protein C and protein S levels at the time of COVID-19 diagnosis and their relation to infection severity.

## Methods

This is a case–control study measuring protein C and S levels in patients infected with COVID-19 compared to the normal population from October 2021 to March 2022.

COVID-19-infected patients were treated in isolation hospitals and outpatient clinics of Ain Shams University Hospitals, Cairo. The study included one hundred participants, who were divided into 2 groups. Group I included sixty patients infected with COVID-19, and group II included forty normal healthy adults. Group I was subclassified into three subgroups according to disease severity: mild, moderate, and severe COVID-19 infections. Classification of patients was according to the WHO clinical progression scale [11].

The included subjects were > 18 years old, patients diagnosed with COVID-19 infection by PCR, patients not in DIC, and patients not suffering from liver cirrhosis.

The excluded patients were patients on vitamin K antagonist anticoagulation therapy, females on oral contraceptive pills, patients who are suffering from acute or subacute thrombosis, and patients with a family history of recurrent deep venous thrombosis (DVT) or protein C and protein S deficiency.

Data were collected, revised, coded, and entered into the Statistical Package for Social Science (IBM SPSS) version 20. The qualitative data were presented as numbers and percentages while quantitative data were presented as mean, standard deviations, and ranges.

The comparison between the two groups with qualitative data was done by using the chi-square test or Fisher exact test when the expected count in any cell was found less than five.

Independent *t*-test and ANOVA test were used for the comparison of quantitative data with the parametric distribution. Turkey’s test was used as a complementary test with the ANOVA test to find means that are significantly different from each other. The confidence interval was set to 95%, and the margin of error accepted was set to 5%.

Written informed consent has been obtained from all patients before participation. Approval for this study will be obtained by the Institutional Review Board (IRB) of Ain Shams Medical School.

## Results

Our study included sixty cases infected with COVID-19 and forty healthy controls. Twenty-four cases were male, and thirty-six were females with a mean age of 57 years old. For our control group, there were twenty-three males and seventeen females with a mean age of 41 years old. The cases group included ten cases with mild severity, twenty-four cases of moderate severity, and twenty-six cases of severe disease according to the WHO clinical progression scale. All patients with moderate and severe disease received oxygen therapy on admission (twenty-two patients needed ventilation on admission). Ninety percent of patients received anticoagulation after admission to the hospital or examination in the clinic and after the withdrawal of blood samples.

Regarding laboratory data, the patient group data showed statistically significant decreases in the lymphocytic count, monocytes, hemoglobin, platelet, albumin, and calcium in comparison with the control group. While there were statistically significant increases in TLC, neutrophils, renal function, liver enzymes, and CRP for the patient group. INR was higher in the patients’ group, and it was statistically significant. There were no statistical differences between the two groups regarding sodium, potassium, and bilirubin (Table 1).

**Table 1:** Laboratory data of patients and control groups

		**Groups**		***T*-test**	
		**Group I (patients)**	**Group II (control)**	***t***	***P*-value**
**Na**	**Range**	113–152	130–143	− 0.137	0.891
	**Mean ± SD**	137.52 ± 6.84	137.67 ± 3.1		
**K**	**Range**	2.8–6.3	3.8–4.6	0.591	0.556
	**Mean ± SD**	4.2 ± 0.67	4.130 ± 0.23		
**Calcium**	**Range**	7.2–9.9	8–10.5	− 3.580	0.001*
	**Mean ± SD**	8.72 ± 0.5	9.14 ± 0.67		
**Creatinine**	**Range**	0.3–9.2	0.5–1.2	4.176	< 0.001*
	**Mean ± SD**	2.14 ± 1.95	0.84 ± 0.18		
**BUN**	**Range**	7–129	8–32	6.337	< 0.001*
	**Mean ± SD**	42.98 ± 28.87	13.73 ± 4.94		
**ALT**	**Range**	3–143	9–55	2.542	0.013*
	**Mean ± SD**	30.95 ± 28.08	19.33 ± 8.28		
**AST**	**Range**	6–232	12–23	3.887	< 0.001*
	**Mean ± SD**	39.93 ± 35.3	18.15 ± 3.08		
**ALK.P**	**Range**	7.9–406	49–291	− 2.107	0.038*
	**Mean ± SD**	97.1 ± 69.51	128.5 ± 78.04		
**Gamma G T**	**Range**	13–337	7–57	5.294	< 0.001*
	**Mean ± SD**	84 ± 68.43	26.08 ± 11.69		
**Albumin**	**Range**	1.6–4	3.5–5.3	− 15.339	< 0.001*
	**Mean ± SD**	2.96 ± 0.54	4.56 ± 0.46		
**Total bilirubin**	**Range**	0.2–2.3	0.3–1.09	− 0.428	0.670
	**Mean ± SD**	0.69 ± 0.41	0.72 ± 0.22		
**Direct bilirubin**	**Range**	0–1.2	0.09–0.37	1.391	0.167
	**Mean ± SD**	0.25 ± 0.23	0.2 ± 0.07		
**TLC**	**Range**	3.8–25.2	4.3–9.4	4.379	< 0.001*
	**Mean ± SD**	9.73 ± 4.52	6.49 ± 1.44		
**Neut**	**Range**	2.6–20.1	1.81–6.8	5.889	< 0.001*
	**Mean ± SD**	8.15 ± 4.17	4.12 ± 1.37		
**Lymph**	**Range**	0.2–5.2	0.5–2.81	− 3.874	< 0.001*
	**Mean ± SD**	1.19 ± 0.894	1.84 ± 0.7		
**Mono**	**Range**	0–1.1	0–1	− 2.359	0.020*
	**Mean ± SD**	0.35 ± 0.27	0.48 ± 0.24		
**Hgb**	**Range**	7.6–17.7	10.3–17	− 6.153	< 0.001*
	**Mean ± SD**	11.57 ± 2.19	14.07 ± 1.64		
**PLT**	**Range**	41–586	202–417	− 3.803	< 0.001*
	**Mean ± SD**	220.55 ± 104.28	288.48 ± 52.84		
**CRP**	**Range**	0.1–327	0.1–5	7.175	< 0.001*
	**Mean ± SD**	83.79 ± 71.42	2.63 ± 1.3		
**INR**	**Range**	0.9–3.7	0.8–1.2	3.260	0.002*
	**Mean ± SD**	1.22 ± 0.39	1.02 ± 0.08		

^*^Statistically significant difference

**Fig. 1:**
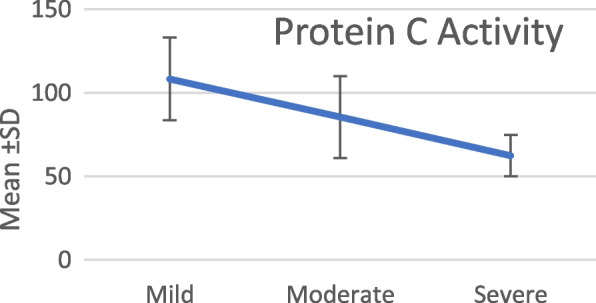
Serum protein C in different disease severity groups

The activity of protein C in the patient group serum was significantly lower than the control group serum (79.35 ± 26.017 vs 97.43 ± 15.007; *p* < *0.001*). Protein S is also significantly decreased in the patient group serum when compared to the control group (70.233 ± 22.476 vs 91 ± 14.498; *p* < *0.001*) (Table 2, Fig. 1).

**Table 2:** Protein C and S activity difference between the study groups

		**Groups**		***T*-test**	
		**Group I (patients)**	**Group II (control)**	***t***	***P*-value**
**Protein C**	**Range**	42–138	44–127	− 3.972	< 0.001*
	**Mean ± SD**	79.35 ± 26.02	97.43 ± 15.01		
**Protein S**	**Range**	35–113	0.8–1.2	− 5.166	< 0.001*
	**Mean ± SD**	70.23 ± 22.48	91 ± 14.5		

^*^Statistically significant difference

**Fig. 2:**
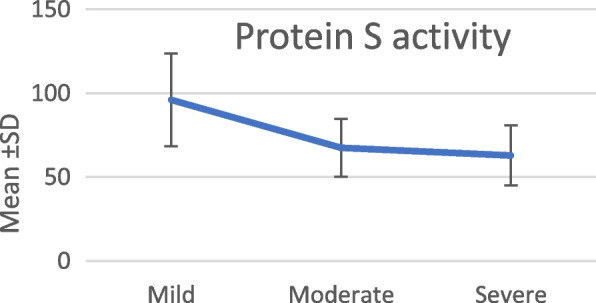
Serum protein S in different disease severity groups

It is shown that the levels of protein C activity and protein S activity in the serum were a statistically significant decrease in relation to the increase in the disease severity (*p* < *0.001*). However, in protein S, this significance is mainly due to the difference between the mild and the severe group (*p* < *0.001*), the mild and moderate group (*p* < *0.001*) with no significant difference between the moderate and severe groups (*p* 0.704) (Table 3, Fig. 2).

**Table 3:** Protein C and S activity in different COVID-19 severity cases

	**Disease severity**			**ANOVA test**		**Tukey’s test**		
	**Mild, mean ± SD**	**Moderate, mean ± SD**	**Severe, mean ± SD**	***F***	***P*-value**	**MI vs MO**	**MI vs S**	**MO vs S**
**Protein C**	108.4 ± 24.72	85.58 ± 24.43	62.42 ± 12.47	20.74	< 0.001*	0.011*	< 0.001*	< 0.001*
**Protein S**	96.00 ± 27.63	67.37 ± 17.2	62.96 ± 17.8	10.84	< 0.001*	< 0.001*	< 0.001*	0.704

^*^Statistically significant difference

## Discussion

From the early days of the COVID-19 pandemic in Wuhan, China, the risk of thrombosis among patients infected with COVID-19 was reported [5].

COVID-19-associated coagulopathy (CAC) is one of the leading causes of morbidity and mortality in patients infected with COVID-19 [12]. CAC can lead to the formation of microthrombi and macrothrombi, resulting in end organ damage, including the lungs, heart, brain, and kidneys [8].

Many reports suggested different mechanisms for CAC which include complex dysregulated interactions between the inflammatory, immune, coagulation, fibrinolytic, complement, and kallikrein-kinin systems [13–16].

This study aimed to assess protein C and S levels in COVID-19-infected patients at the time of diagnosis and their relation to infection severity.

Our study found a significant age difference between the groups as the age increased with the increase in disease severity which is like what was reported in a previous study [17].

It showed that serum levels of protein C and protein S were a statistically significant decrease in the COVID-19-infected patients compared to the control group, and this comes in agreement with Corrêa et al. who assessed the coagulation profile of patients with COVID-19 admitted to the ICU [18].

Comparing protein C and protein S activity levels in different disease severity, our study revealed a statistically significant decrease in both protein levels with the increase in the disease severity. The decrease in protein C level was significant across the three different disease severity subgroups. However, for protein S, the significance was between the mild group when compared to the moderate and the severe group, but there was no significant difference between the moderate group and the severe group.

This came in accordance with Corrêa et al.’s study which was performed on thirty ICU patients. However, they divided patients into 2 groups according to SOFA score with one group SOFA ≤ 10 and the other SOFA > 10 and showed there were decreases in both protein C and protein S in patients in both groups. However, this decrease was more in patients with SOFA > 10 [18].

Another study was performed on 231 COVID-19 hospitalized patients with moderate to severe illness (according to the WHO COVID-19 outcomes scale) and assessed admission plasma levels of 12 hemostatic proteins and their association with the disease severity and the disease outcome. It concluded that lowered admission plasma levels of protein C in patients with COVID-19 presenting with moderate or severe illness may be a useful biomarker for disease morbidity and mortality [19].

Stoichitoiu and his group assessed admission protein S in relation to disease severity and disease outcome. They found that protein S activity was lower in patients with COVID-19, and its level was associated with disease severity and mortality, suggesting that it may have a role in the thromboembolism associated with COVID-19 infection [20].

To conclude, the admission levels of protein C and protein S activities are decreased in COVID-19-infected patients when compared to the healthy population. It also, concluded that the decrease in their levels is in statistically significant relation to the disease severity in COVID-19-infected patients. Clinical trials to assess the impact of using activated protein C in patients with COVID-19 are highly recommended, especially in patients with severe disease.

## Data Availability

All the data and materials used in this research are available upon request from the corresponding author.

## Abbreviations

CAC: COVID-19-associated coagulopathy
DIC: Disseminated intravascular coagulation
DVT: Deep venous thrombosis
ICU: Intensive care unit
VTE: Venous thromboembolism
